# Novel Bidirectional Output Ytterbium-Doped High Power Fiber Lasers: From Continuous to Quasi-Continuous

**DOI:** 10.3390/mi15010153

**Published:** 2024-01-20

**Authors:** Lingfa Zeng, Xinyi Ding, Jiaqi Liu, Xiaolin Wang, Yun Ye, Hanshuo Wu, Peng Wang, Xiaoming Xi, Hanwei Zhang, Chen Shi, Fengjie Xi, Xiaojun Xu

**Affiliations:** 1 College of Advanced Interdisciplinary Studies, National University of Defense Technology, Changsha 410073, China; zenglingfa14@sina.com (L.Z.); chinaxyding@163.com (X.D.); liujiaqi_@nudt.edu.cn (J.L.); yeyun2015@163.com (Y.Y.); whsopt@126.com (H.W.); wangpeng13@nudt.edu.cn (P.W.); exixiaoming@163.com (X.X.); zhanghanwei100@163.com (H.Z.); bigbryant@nudt.edu.cn (C.S.); xifengjie@163.com (F.X.); xuxj@21cn.com (X.X.); 2 Nanhu Laser Laboratory, National University of Defense Technology, Changsha 410073, China; 3 Hunan Provincial Key Laboratory of High Energy Laser Technology, National University of Defense Technology, Changsha 410073, China

**Keywords:** bidirectional output ytterbium-doped high-power fiber lasers, quasi-continuous wave fiber laser, stimulated Raman scattering, transverse mode instability

## Abstract

Traditional ytterbium-doped high-power fiber lasers generally use a unidirectional output structure. To reduce the cost and improve the efficiency of the fiber laser, we propose a bidirectional output fiber laser (BOFL). The BOFL has many advantages over that of the traditional unidirectional output fiber laser (UOFL) and has a wide application in the industrial field. In theory, the model of the BOFL is established, and a comparison of the nonlinear effect in the traditional UOFL and the BOFL is studied. Experimentally, high-power continuous wave (CW) and quasi-continuous wave (QCW) BOFLs are demonstrated. In the continuous laser, we first combine the BOFL with the oscillating amplifying integrated structure, and a near-single-mode bidirectional 2 × 4 kW output with a total power of above 8 kW is demonstrated. Then, with the simple BOFL, a CW bidirectional 2 × 5 kW output with a total power of above 10 kW is demonstrated. By means of pump source modulation, a QCW BOFL is developed, and the output of a near-single mode QCW laser with a peak output of 2 × 4.5 kW with a total peak power of more than 9 kW is realized. Both CW and QCW output BOFL are the highest powers reported at present.

## 1. Introduction

Thanks to their convenient thermal management, flexible transmission, high efficiency, and good beam quality, high-power fiber lasers have been widely used, especially in industrial, medical, and scientific research fields [1,2,3]. The rapid development of active fibers, passive fiber devices, and high-brightness pump sources has ushered in a golden period for the development of high-power fiber lasers in the past two decades. In the entire fiber laser industry, the IPG company has always been in a leading position. As early as 2009, they launched a nearly-single-mode 10 kW fiber laser [4]. With years of development, multiple research institutions have accumulated rich experience and achieved fruitful results in the field of fiber lasers [5,6,7,8,9,10,11,12]. Before the discovery of transverse mode instability (TMI), nonlinear effects represented by stimulated Raman scattering (SRS) were the main limiting factors for the increase in the output power of high-power fiber lasers [13]. The widespread use of large-mode field area fibers has effectively suppressed the nonlinear effects. However, as the output power further increases, TMI has been discovered in high-power fiber lasers [14,15,16,17,18,19,20,21,22,23,24,25,26,27,28,29,30,31,32,33,34]. TMI in fibers originates from thermal effects caused by quantum defects, photon darkening, and other factors in active fibers [16,17,23,24]. Under the influence of the mode interference pattern and thermal optical effect, long-period refractive index fiber gratings appear in the fiber core. When the phase matching condition is met between the mode interference pattern and the long-period refractive index grating, mode coupling occurs between the fundamental mode and higher-order mode, resulting in TMI. The emergence of TMI is often accompanied by beam quality degradation and chaotic output timing, seriously endangering device safety [22,25]. So far, SRS and TMI have become the main factors limiting the power increase of high-power fiber lasers, and there is a sharp contradiction in their suppression methods [35,36]. Researchers have conducted extensive theoretical and experimental research on the suppression of SRS and TMI, including active fiber, signal wavelength, pump wavelength, pump configuration, pump modulation, and fiber coiling [37,38,39,40,41,42,43,44,45]. With the support of the above suppression measures, the output power of fiber lasers has achieved great breakthroughs. At present, fiber laser amplifiers with an output power of over 10 kW are relatively mature, and the output power of a single fiber has exceeded 20 kW [5,6,46]. The output power of the all-fiber structure fiber laser oscillator has also exceeded 8 kW [9,11]. From the current situation, further improvement of the output power of fiber lasers still faces considerable difficulties.

Traditional fiber lasers, whether fiber amplifiers based on MOPA or fiber oscillators, have only one output port and belong to unidirectional output fiber lasers (UOFL). For oscillators, because the reflectivity of actual high reflectivity gratings cannot reach 100%, there is always a portion of light leaking from one side of the high reflectivity grating. This leaked light is difficult to utilize, and the processing is complex. In 2022, we proposed a novel linear cavity bidirectional output fiber laser oscillator [47]. This structure uses a low reflectivity grating instead of a high reflectivity grating to achieve bidirectional laser output. Compared with other laser structures that achieve bidirectional output, this structure can achieve a stable high-power output and has extremely good application prospects. Compared with traditional fiber lasers, bidirectional output fiber lasers can achieve two laser outputs based on a single resonant cavity structure, reducing the number of devices, simplifying cooling devices and control systems, and compressing the volume and weight of the system while reducing system costs. In addition, based on bidirectional output, a single fiber laser with higher output power can be achieved through power combining. From the power distribution in the fiber, this structure has better nonlinear effect suppression ability than unidirectional output fiber lasers, which is more conducive to improving output power. Whether in industrial fields represented by laser cutting and welding, or in high-power beam synthesis fields that require high compactness of laser systems, bidirectional output fiber lasers are superior.

Despite the mature development of fiber lasers, improving output power has become increasingly difficult, and in many fields, CW fiber lasers generate a large amount of waste energy. Compared with continuous fiber lasers, quasi-continuous wave (QCW) fiber lasers have the advantages of an adjustable repetition frequency and pulse width, high temporal stability, high electro–optical conversion efficiency, and high peak power [48]. They have significant advantages in special material cutting, precision welding, micro drilling, and other fields such as medicine and aerospace. Since 2013, QCW fiber lasers have experienced rapid development, and mature products have been launched [48,49,50]. However, the peak power of lasers that maintain near single-mode beam quality is below 3 kW, and the beam quality is relatively poor in high-power states above 3 kW. From 2022 to 2023, researchers from the University of National Defense Technology reported on high beam quality QCW fiber lasers with a power of 6–10 kW [49,50,51]. At a peak power of 10.75 kW, the beam quality remained at ~1.6 [51]. In addition, according to existing reports, QCW fiber lasers have the potential to suppress TMI.

This article is based on a bidirectional output fiber laser and introduces the bidirectional output fiber laser in both CW and QCW operating states, including continuous wave bidirectional output fiber oscillators (CW BOFL-OS), continuous wave bidirectional output oscillating amplifying integrated fiber lasers (CW BOFL-OA), quasi-continuous wave bidirectional output fiber oscillators (QCW BOFL-OS), and QCW + CW bidirectional output fiber oscillators (QCW + CW BOFL-OS). Firstly, a theoretical model of a BOFL based on the rate equation system was established, and on this basis, the suppression effect of the BOFL on the nonlinear effects in the fiber laser was analyzed. Subsequently, it was experimentally verified that the BOFL has better SRS and TMI suppression capabilities compared to UOFL. By integrating the optimization measures of SRS and TMI, 2 × 2–5 kW CW BOFL-OS and 2 × 2–4 kW CW BOFL-OA were achieved in sequence. On the basis of the CW fiber laser, the QCW BOFL-OS has been expanded, and a 2 × 4.5 kW laser output has been achieved. Finally, a new method to suppress TMI using the QCW + CW operation mode was introduced. The results in the article can provide guidance for the development of high-power fiber lasers.

## 2. Theory of a BOFL and Comparison with a UOFL

### 2.1. Structure and Theory of BOFL

**Figure 1:**
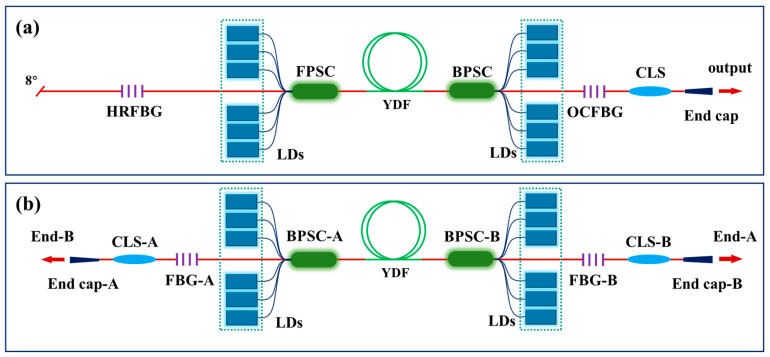
Comparison diagram of the structure of the UOFL and BOFL-OS. (**a**) Schematic diagram of the UOFL.; (**b**) schematic diagram of the BOFL-OS.

Figure 1 shows a schematic diagram of the structure of a bidirectional output fiber laser oscillator (BOFL-OS). Structurally, a BOFL-OS has high compatibility with a UOFL. As shown in Figure 1a, the resonant cavity of a UOFL is usually composed of a high reflectivity fiber Bragg grating (HRFBG) and an output coupler fiber Bragg grating (OCFBG). In an ideal state, the reflectivity of the HRFBG is 1. When the signal is transmitted to the HRFBG, it is all reflected into the active fiber for further amplification. The reflectivity of the OCFBG is less than 1. When the signal light is transmitted to the OCFBG, part of it is reflected into the active fiber for further amplification, and the other part is transmitted through the OCFBG to become the output laser. However, in actual production, the HRFBG cannot reflect 100% of the signal back to the resonant cavity. Part of the laser will be transmitted through the HRFBG, known as backward light. The strength of the backward light is closely related to the actual reflectivity of the HRFBG. The lower the actual reflectivity of the HRFBG, the higher the intensity of the backward light. If the cladding light stripper (CLS) and end cap are sequentially fused in the direction of the backward light, the UOFL is converted into the BOFL-OS, as shown in Figure 1b. When the reflectivity of two FBGs is equal, the signal output from both ends is also equal. The BOFL-OS we mentioned later uses two OCFBGs to form a resonant cavity. In addition, for ease of description, the output end of the BOFL-OS is distinguished into end A and end B. When applying a unidirectional pump configuration, turning on the pump source close to end A (B) is called end A (B) pumping. When conducting comparative experiments between the UOFL and BOFL-OS, the end B is equivalent to the laser output end of the UOFL.

Based on the rate equation model of fiber lasers, we have established a theoretical model of a BOFL-OS and conducted a theoretical analysis of them. The theoretical model of a BOFL-OS with a multi-pump and signal wavelength, considering ASE, is as follows.

(1) $$\begin{array}{l} \pm \frac{dP_{n}^{s\pm }\left(\lambda_{n}^{s},z,t\right)}{dz}=\pm \frac{\partial P_{n}^{s\pm }\left(\lambda_{n}^{s},z,t\right)}{\partial z}+\frac{1}{v_{s}}\frac{\partial P_{n}^{s\pm }\left(\lambda_{n}^{s},z,t\right)}{\partial t}\\ \;=\Gamma_{n}^{s}\left(\lambda_{n}^{s}\right)\left[\sigma_{n}^{es}\left(\lambda_{n}^{s}\right)N_{2}\left(z,t\right)-\sigma_{n}^{as}\left(\lambda_{n}^{s}\right)N_{1}\left(z,t\right)\right]P_{n}^{s\pm }\left(\lambda_{n}^{s},z,t\right)\\ \;+2\sigma_{n}^{es}\left(\lambda_{n}^{s}\right)N_{2}\left(z,t\right)\frac{hc^{2}}{{\left(\lambda_{n}^{s}\right)}^{3}}\Delta \lambda -\alpha_{n}^{s}\left(\lambda_{n}^{s},LP_{k}\right)P_{n}^{s\pm }\left(\lambda_{n}^{s},LP_{k},z,t\right)\\ \;+\Gamma_{n}^{s}\left(\lambda_{n}^{s}\right)P_{n}^{s\pm }(\lambda_{n}^{s},z,t)\sum_{i=1}^{N}\frac{1}{A_{eff}^{i,n}}g_{R}(\omega_{i}-\omega_{n})\left[P_{i}^{s+}(\lambda_{i}^{s},z,t)+P_{i}^{s-}(\lambda_{i}^{s},z,t)\right] \end{array}$$

(2) $$\begin{array}{l} \pm \frac{dP_{m}^{p\pm }\left(\lambda_{m}^{p},z,t\right)}{dz}=\pm \frac{\partial P_{m}^{p\pm }\left(\lambda_{m}^{p},z,t\right)}{\partial z}+\frac{1}{v_{p}}\frac{\partial P_{m}^{p\pm }\left(\lambda_{m}^{p},z,t\right)}{\partial t}\\ \;=\Gamma_{m}^{p}\left(\lambda_{m}^{p}\right)\left[\sigma_{m}^{ep}\left(\lambda_{m}^{p}\right)N_{2}\left(z,t\right)-\sigma_{m}^{ap}\left(\lambda_{m}^{p}\right)N_{1}\left(z,t\right)\right]P_{m}^{p\pm }\left(\lambda_{m}^{p},z,t\right)\\ \;-\alpha_{m}^{p}\left(\lambda_{m}^{p}\right)P_{m}^{p\pm }\left(\lambda_{m}^{p},z,t\right) \end{array}$$

(3) $$\begin{array}{l} \frac{\partial N_{2}(z,t)}{\partial t}=\frac{1}{hc}\sum_{m=1}^{M}\frac{\Gamma_{m}^{p}\left(\lambda_{m}^{p}\right)}{A_{m}^{p}\left(\lambda_{m}^{p}\right)}\lambda_{m}^{p}\left[\sigma_{m}^{ap}\left(\lambda_{m}^{p}\right)N_{1}(z,t)-\sigma_{m}^{ep}\left(\lambda_{m}^{p}\right)N_{2}(z,t)\right]\left[P_{m}^{p+}\left(\lambda_{m}^{p},z,t\right)+P_{m}^{p-}\left(\lambda_{m}^{p},z,t\right)\right]\\ \begin{array}{c} \;+\frac{1}{hc}\sum_{n=1}^{N}\frac{\Gamma_{n}^{s}\left(\lambda_{n}^{s}\right)}{A_{n}^{s}\left(\lambda_{n}^{s}\right)}\lambda_{n}^{s}\left[\sigma_{n}^{as}\left(\lambda_{n}^{s}\right)N_{1}(z,t)-\sigma_{n}^{es}\left(\lambda_{n}^{s}\right)N_{2}(z,t)\right]\left[P_{n}^{s+}\left(\lambda_{n}^{s},z,t\right)+P_{n}^{s-}\left(\lambda_{n}^{s},z,t\right)\right] \end{array}\\ \;-\frac{N_{2}(z,t)}{\tau } \end{array}$$

(4) $$N\left(z,t\right)=N_{1}\left(z,t\right)+N_{2}\left(z,t\right).$$

Equations (1) and (2) describe the power distribution of the signal and the pump in the active fiber, respectively. Equations (3) and (4) describe the distribution of the number of particles in excited and ground states in the fibers.

In Equation (1), $g_{R}(\omega_{i}-\omega_{n})$ refers to the Raman gain of the nth signal wavelength received by the *i*-th wavelength (if this value is negative, it is a loss), and its expression is:

(5) gR(ωi−ωn)=43γkfRIm{h˜R(ωi−ωn)}.

In the formula, ${\tilde{h}}_{R}(\Delta \omega_{i})$ is the Fourier transform of the Raman response function $h_{R}(t)$ :

(6) $$h_{R}(t)=\left\{\begin{array}{ll} \frac{\tau_{1}^{2}+\tau_{2}^{2}}{\tau_{1}\tau_{2}^{2}}e^{-t/\tau_{2}}\sin(\frac{t}{\tau_{1}}), & t\geq 0\\ 0, & t<0 \end{array}\right.$$

(7) $${\tilde{h}}_{R}(\Delta \omega )=\frac{1}{2i}\frac{\tau_{1}^{2}+\tau_{2}^{2}}{\tau_{1}\tau_{2}^{2}}\left\{\frac{1}{\frac{1}{\tau_{2}}-i(\Delta \omega +\frac{1}{\tau_{1}})}-\frac{1}{\frac{1}{\tau_{2}}-i(\Delta \omega -\frac{1}{\tau_{1}})}\right\}.$$

Consider the total Raman gain of the nth signal wavelength for all signal wavelengths:

(8) $$G_{R\_n}^{net}=\sum_{i=1}^{N}\frac{g_{R}(\omega_{i}-\omega_{n})}{A_{eff}^{\left(i,n\right)}}P_{si},$$

where $A_{eff}^{\left(i,n\right)}$ is the effective mode field area between the *i*-th and *n*-th signal wavelengths, usually replaced by the effective core area $A_{eff}$ . The main physical quantities and their physical meanings appearing in the rate equation are shown in Table 1.

**Figure 2:**
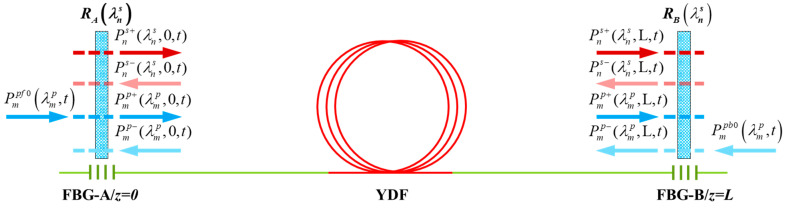
Simplified structure of a BOFL.

Figure 2 is a simplified structure of a BOFL-OS. Due to the conversion of the pump light to signal light occurring within the active fiber, the resonant cavity of this model takes a section of active fiber with a length of L, defined as the leftmost end z = 0 and the rightmost end z = L. The pump and signal transmitted from left to right are the forward transmission, while the pump and signal transmitted from right to left are the backward transmission. The transmission of the forward signal $P_{n}^{s+}(\lambda_{n}^{s},z,t)$ and backward signal $P_{m}^{p-}(\lambda_{m}^{p},z,t)$ in the resonant cavity satisfies the rate Equation (9). $R_{a}(\lambda_{n}^{s})$ and $R_{b}(\lambda_{n}^{s})$ are the reflectivity of the fiber Bragg gratings located on both sides of the resonant cavity for the signal light of different wavelengths, and it is generally believed that the fiber Bragg gratings do not reflect the pump light. Therefore, the signal and pump boundary conditions of the BOFL can be obtained as follows:

(9) $$\left\{\begin{array}{l} P_{n}^{s+}\left(\lambda_{n}^{s},0,t\right)=P_{n}^{s-}\left(\lambda_{n}^{s},0,t\right)R_{A}\left(\lambda_{n}^{s}\right)\\ P_{n}^{s-}\left(\lambda_{n}^{s},L,t\right)=P_{n}^{s+}\left(\lambda_{n}^{s},L,t\right)R_{B}\left(\lambda_{n}^{s}\right) \end{array}\right.$$

(10) $$\left\{\begin{array}{l} P_{m}^{p+}\left(\lambda_{m}^{p},0,t\right)=P_{m}^{pf0}\left(\lambda_{m}^{p},t\right)\\ P_{m}^{p-}\left(\lambda_{m}^{p},L,t\right)=P_{m}^{pb0}\left(\lambda_{m}^{p},t\right) \end{array}\right..$$

The reflectivity $R_{A}(\lambda_{n}^{s})$ and $R_{B}(\lambda_{n}^{s})$ of the fiber Bragg grating is a parameter that needs to be set. If the reflectivity of FBG-A to the center wavelength of the signal light is set to a value close to 1, such as 0.99, and the reflectivity of FBG-B to the center wavelength of the signal light is set to a value less than 1, such as 0.1, then the laser can be regarded as a UOFL from the right boundary. The A (B) pumping of the original BOFL-OS is equivalent to the forward (backward) pump of the UOFL. For a BOFL, the reflectivity at both ends is set to a value less than 1. When the parameters of the gratings at both ends are set uniformly, the output power at both ends is consistent.

### 2.2. Theoretical Simulation and Comparison of BOFL and UOFL

For CW fiber lasers, theoretical analysis using steady-state rate equations is a more effective approach. Therefore, based on the theoretical model in Section 2.1, it is simplified as a steady-state rate equation system for theoretical analysis of a CW BOFL. Here, we will mainly compare the nonlinear effects and temperature distribution in a BOFL and UOFL. When comparing the nonlinear effects of the two structures, in addition to comparing and analyzing the spectra calculated by the theoretical model, we also calculated the B-integral based on the calculated power distribution curve and fiber parameters, which can be used to evaluate the nonlinear effects in the fiber. The formula is as follows:

(11) $$B\left(z\right)=\frac{2\pi }{\lambda }\int n_{2}\frac{P\left(z\right)}{A_{eff}}dz.$$

Among them, $n_{2}=2.6\times 10^{-20}\;m^{2}/W$ , is the nonlinear refractive index during laser transmission. The main parameters in the simulation are shown in Table 2.

#### 2.2.1. Comparison of Power Distribution

**Figure 3:**
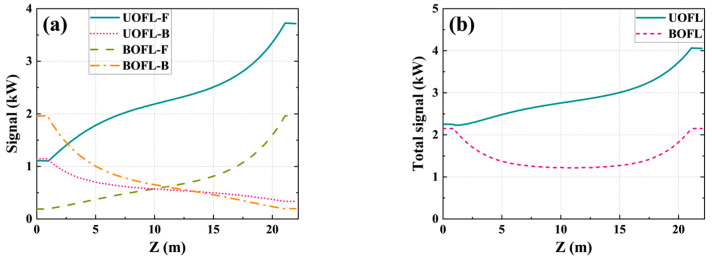
Comparison of power distribution in the BOFL and UOFL under a bidirectional pumping configuration. (**a**) Simulated power distribution; (**b**) total power distribution inside the cavity.

Compared with a UOFL, the most intuitive change brought about by the change in grating reflectivity in a BOFL is the power distribution in the active fiber. As shown in Figure 3, we theoretically calculated the power distribution in the BOFL and UOFL under the same pumping conditions. Among them, Figure 3a shows the forward and backward transmission signal power in the two structures, respectively. For the BOFL, the forward transmission signal represents transmission from end A to end B. In the BOFL, the distribution of signal transmitted in two directions exhibits a symmetrical feature, with an output power of 1763 W at both ends and a total power of 3526 W. In the UOFL, the output laser power is mainly at the output end, which was 3409 W. The BOFL has higher efficiency than the UOFL. Figure 3b shows the total power distribution of the signal inside the active fiber. Under the condition of achieving higher total output power, the BOFL had a lower power density in the active fiber, which was more favorable for the long-term stable operation of the laser.

#### 2.2.2. Comparison of the B-Integral and SRS in a BOFL and UOFL

**Figure 4:**
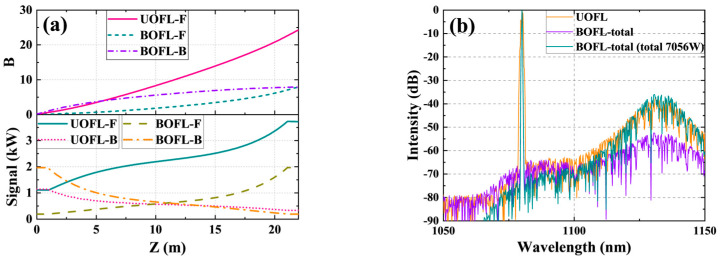
Power distribution, B-integral, and output spectra in the UOFL and BOFL under bidirectional pumping conditions. (**a**) Power distribution and B-integral when applying bidirectional pump configuration with 2000 W power on both ends; (**b**) output spectrum. UOFL: output signal of UOFL. BOFL-total: total output signal of the BOFL when applying bidirectional pump configuration with 2000 W power on both ends. BOFL-total (total 7056 W): total output signal of the BOFL when applying bidirectional pump configuration with 4000 W power on both ends.

Subsequently, we compared and analyzed the SRS and nonlinear effects (B-integral) in the fiber of the BOFL and UOFL when applying a bidirectional pump. Figure 4a shows the power distribution curves and the calculated B-integral in the UOFL and BOFL. From the power distribution, unlike the UOFL, the output power curve of the BOFL has symmetry in the longitudinal distribution, and overall, the distribution is more uniform. From the perspective of the B-integral, the B-integral value of the laser of the UOFL was 27.81, while the B-integral values of the laser of the two ports of the BOFL were 9.72 and 9.73, respectively. To demonstrate the inhibitory effect of the BOFL more intuitively on SRS, we simulated the output spectrum of the BOFL at a total pump power of 8000 W (with a pump power at both ends of 4000 W) based on Figure 4a. Currently, the output power of one end of the BOFL is close to the output power of the UOFL. The result is shown in Figure 4b. When the pumping conditions are completely consistent, i.e., a total pumping power of 4000 W (2000 W on each end), the SRS intensity in the output spectrum of the BOFL was 17.4 dB lower than that in the UOFL. When the single output power level is consistent, the SRS intensity in the output spectrum of the BOFL at a total output power of 7056 W was close to that in the UOFL with an output power of 3409 W. The Raman power in the output laser in Table 3 more clearly demonstrates the suppression advantage of the BOFL for the SRS. Therefore, the BOFL has better nonlinear suppression ability than the UOFL, which helps to improve the output power of lasers in laser systems that are not limited by TMI.

#### 2.2.3. Comparison of Temperature Distribution

**Figure 5:**
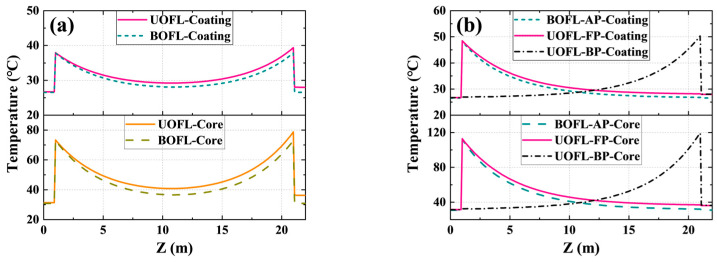
Temperature distribution in the UOFL and BOFL under different pump configurations. (**a**) Temperature distribution under bidirectional pump (2000 W × 2); (**b**) temperature distribution under unidirectional pump (total pump: 4000 W). BOFL-AP: A pumping BOFL. UOFL-FP: forward pump UOFL. UOFL-BP: backward pump UOFL.

Another advantage of the BOFL over the UOFL is the thermal effect in the fibers. Figure 5 shows the temperature distribution of the fiber core and the temperature distribution of the coating in both the BOFL and UOFL under the same conditions (bidirectional pump configuration with 2000 W power on both ends). Among them, the ambient temperature was set to 25 °C, and the system heat transfer coefficient was set to 3000 W/(m^2^K). Although there was a significant difference in the power distribution shown in Figure 4a, the total power in both directions was considered in the temperature calculation, resulting in a similar temperature distribution, as shown in Figure 5. It can be clearly seen that both the core and coating had a lower temperature in the BOFL, indicating a weaker thermal effect in the BOFL. The lower temperature makes the laser safer in practical use, and the reduction of thermal effects is also beneficial for suppressing TMI in fiber lasers, which is more conducive to improving output power. Table 4 shows the maximum temperature calculated under several different pumping configurations. Although there is not much difference in the maximum temperature of the core and cladding of active fibers in the BOFL and UOFL under the same conditions, the overall temperature in the BOFL is slightly lower than that in the UOFL, with significant advantages.

#### 2.2.4. Summary

This section first compares the differences in power distribution between a BOFL and UOFL. Under the same total output power conditions, the BOFL has a lower power density, which is more conducive to the long-term stable operation of the laser. Meanwhile, we verify that the BOFL has a better nonlinear effect suppression effect than the UOFL under the same conditions based on the theoretical model and B-integral. Meanwhile, the maximum temperature of the core and coating of the BOFL is lower than that of the UOFL under the same conditions. Considering the relationship between the TMI and temperature distribution in the fibers, this may be beneficial for suppressing the TMI. Therefore, the BOFL has the potential to balance TMI and SRS, which is more conducive to improving the output power.

### 2.3. Comparison and Verification of the BOFL and UOFL

#### 2.3.1. Comparison of SRS Effect in the BOFL and UOFL in QCW States

**Figure 6:**
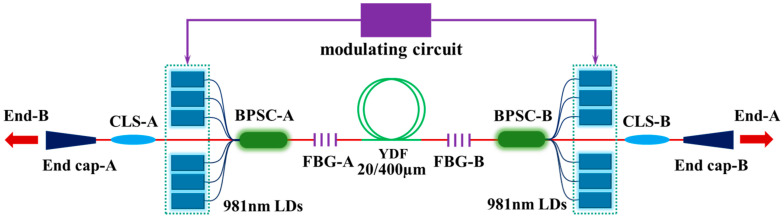
Structural diagram of the QCW BOFL-OS.

We compared the SRS in the BOFL and UOFL based on a QCW fiber laser oscillator. Figure 6 shows a schematic diagram of the structure of a QCW BOFL-OS. A DCYDF with a core-cladding diameter of 20/400 µm was used as the active fiber for the laser. The total length of the fiber was 25 m, and the absorption coefficient for the pump light with a center wavelength of 981 nm was 0.61 dB/m. The center wavelengths of the two gratings were 1079.94 nm and 1079.97 nm, respectively. The 3 dB bandwidths were 0.99 nm and 0.98 nm, and the reflectivity was 10.6% and 10.9%, respectively. The laser uses a pump source with a central wavelength of 981 nm. All pump sources were operating in the QCW mode; the modulation frequency of the pump source was 1 kHz, and the pulse duty cycle was 10%.

**Figure 7:**
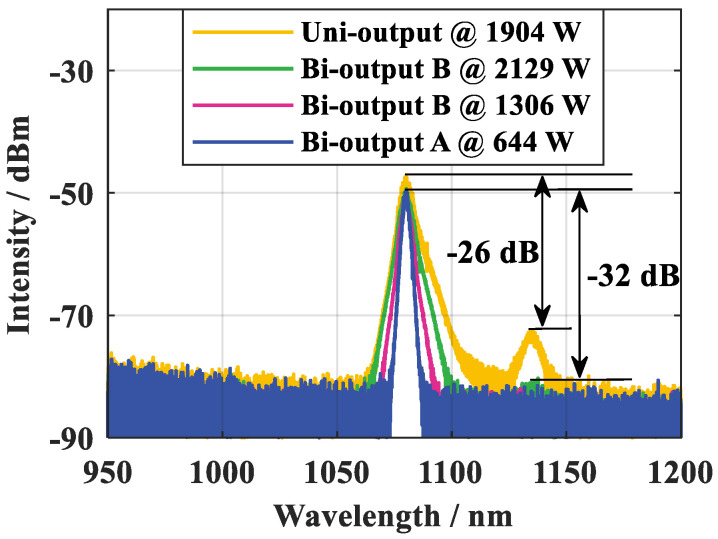
Comparison of output spectra between the QCW UOFL and QCW BOFL [52].

Figure 7 shows a comparison of the output spectra of the QCW UOFL and QCW BOFL. In the QCW BOFL, the pump configuration is a bidirectional pump, with the maximum average pump power at both ends of 239.6 W and 258.1 W, respectively. Currently, the peak output power at end B is 2119 W. There is no obvious SRS in the output spectrum of end B. For the QCW UOFL, a forward pump configuration is adopted, with a maximum average pump power of 266.5 W and a peak output power of 1904 W. The SRS intensity was 26 dB lower than the signal. Intuitively, when the output power of one end of the QCW BOFL was equivalent to that of the QCW UOFL, it also demonstrated better SRS suppression capability. It cannot be denied that the pump configuration had a significant impact on SRS. However, for the output laser of end B in the QCW BOFL, a pump light of 239.6 W was equivalent to a forward pump, and the total peak output power of the QCW BOFL under current conditions reached 3313 W. Therefore, the results fully demonstrated that the BOFL had better SRS inhibition ability compared to UOFL.

#### 2.3.2. Comparison of theTMI effect in the BOFL and UOFL in CW States

**Figure 8:**
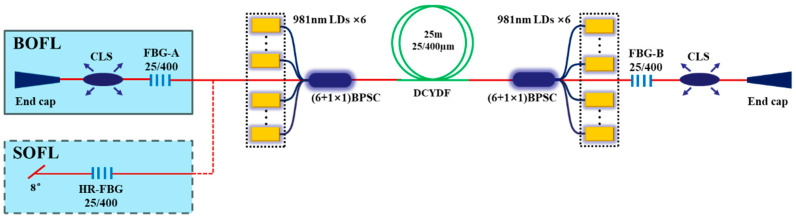
Structural diagram of the TMI threshold comparison experiment between the UOFL and BOFL.

The simulation analysis of temperature in Section 2.2.2 indicates that under the same conditions, a BOFL may have a higher TMI threshold than a UOFL. Therefore, we designed an experiment to compare the TMI thresholds of a UOFL and BOFL under the same experimental conditions. As shown in Figure 8, the laser uses a double-cladding ytterbium-doped fiber with a core-cladding diameter of 25/400 µm and a total length of 25 m. The cladding pump absorption coefficient of the pump light with a central wavelength of 915 nm was about 0.56 dB/m. The reflectivity of the OCFBG (FBG-A and FBG-B) used was about 10%, and the 3 dB bandwidth was ~1.0 nm. The 3 dB bandwidth of the HRFBG in the UOFL was approximately 3.0 nm. The laser uses a wavelength stabilized 981 nm LDs as the pump source, and two pump and signal combiners were fused with six sets of pump sources. Consistent with the simulation in Section 2.2, we strictly compared the TMI threshold of the laser under the same conditions. The pump configuration applied was a bidirectional pump, with a pump power ratio of 1:1 at both ends. The experimental results are shown in Table 5. For the UOFL, its TMI threshold was only 1910 W, while for the BOFL, the TMI threshold could reach 2515 W, which was a 31.7% increase in threshold compared to the UOFL. Therefore, this result indicates that the BOFL has a higher TMI threshold compared to the UOFL.

**Figure 9:**
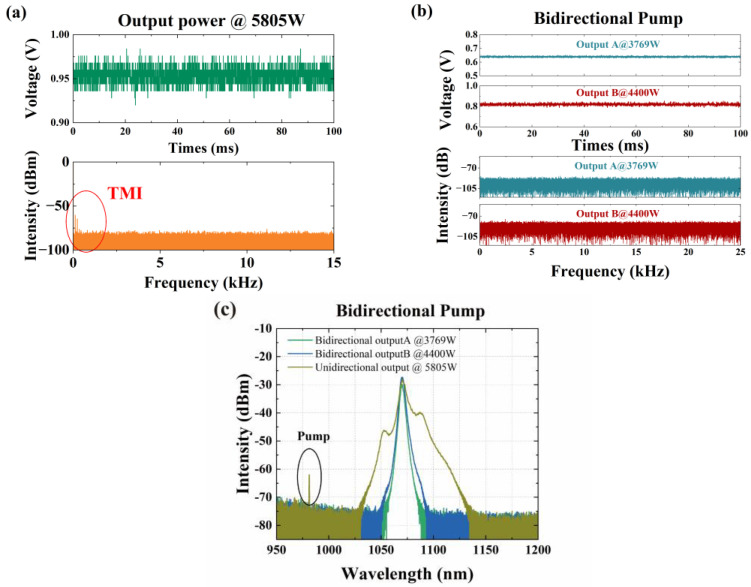
Comparison of experimental results between the UOFL and BOFL based on 30/400 µm double-cladding ytterbium-doped fiber. (**a**) The PD signal and corresponding FFT result of the UOFL at maximum output power; (**b**) the PD signal and corresponding FFT result of the BOFL at maximum output power; (**c**) spectral comparison of the UOFL and BOFL at maximum output power [53].

In 2023, our research group achieved a 2 × 4 kW BOFL based on 30/400 µm double-cladding ytterbium-doped fiber [53]. At the same time, the TMI thresholds of the UOFL and BOFL were compared. As shown in Figure 9, under bidirectional pump conditions and limited by TMI, the maximum output power of the UOFL was only 5805 W. However, the BOFL can achieve laser output above 8 kW without TMI. Meanwhile, from the spectral broadening in Figure 9c, the BOFL had a better nonlinear effect suppression ability than UOFL. This is very useful for the industry power combining employing an all-fiber combiner.

#### 2.3.3. Summary

Through the comparative experiments on the BOFL and UOFL mentioned above, we can see that the BOFL has significant advantages in achieving high-power fiber lasers. For SRS, whether under the same total output power or single-end output power, BOFL has better SRS suppression ability, which is consistent with theoretical simulation results. For TMI, the total output power when the TMI appears in the BOFL is much higher than that when TMI appears in the UOFL. Therefore, the BOFL has the advantage of balancing SRS and TMI, which is more conducive to achieving high-power fiber lasers.

## 3. Experimental Study on Continuous Wave High Average Power BOFL

### 3.1. Experimental Study on High Average Power BOFL-OS

#### 3.1.1. 2–4 kW High-Power BOFL-OS

**Figure 10:**
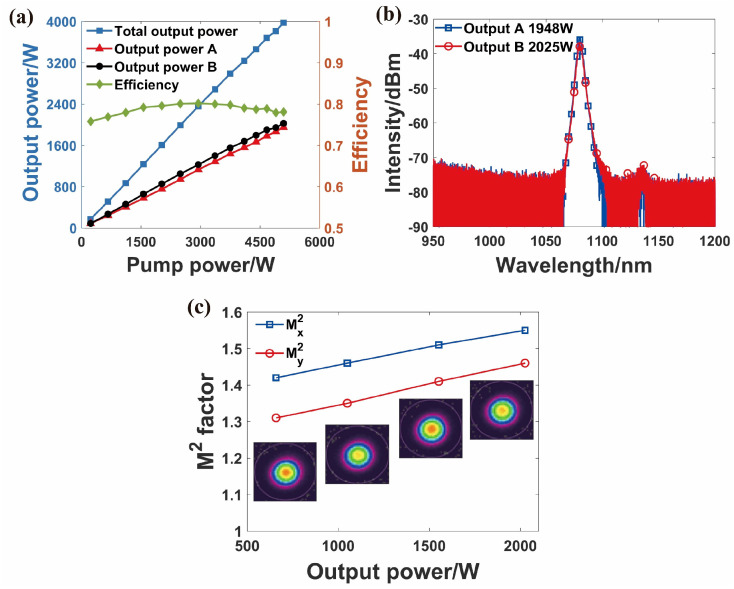
The main output result of the 2 × 2 kW BOFL-OS. (**a**) The output power and efficiency vary with the pump power; (**b**) the output spectrum at the maximum output power; (**c**) evolution of beam quality during the experiment [47].

**Figure 11:**
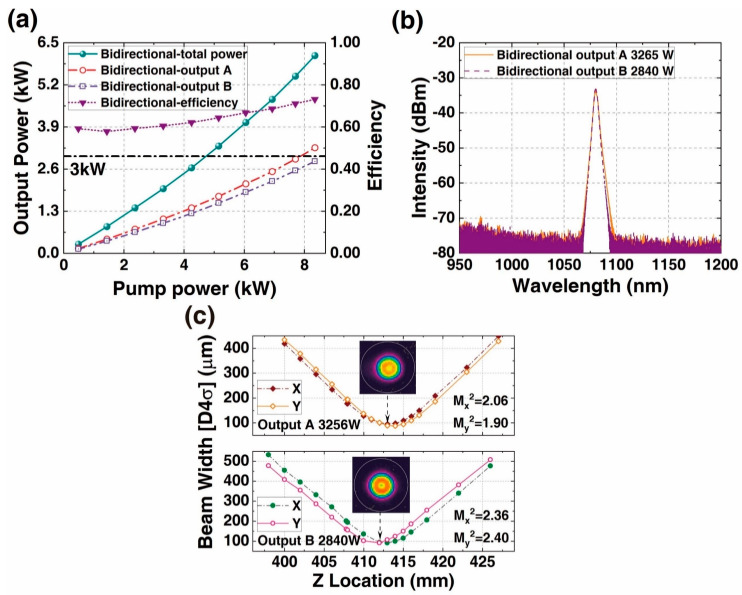
The main results of a 2 × 3 kW BOFL-OS based on spindle-shaped fibers. (**a**) The output power and efficiency vary with the pump power; (**b**) the output spectrum at the maximum output power; (**c**) the beam quality at the maximum output power [54].

In 2022, our research group first proposed a BOFL-OS and achieved a 2×2 kW laser output [47]. The structure of the laser is shown in Figure 1b, using a DCYDF with a core/cladding diameter of 20/400 µm, a pair of OCFBGs with a 3 dB bandwidth of approximately 1 nm, and a wavelength stabilized 976 nm LDs. As shown in Figure 10, at the maximum output power, the power at the two ends is 1948 W and 2025 W, respectively. Due to pump power limitations and potential enhanced SRS, the output power of the laser remained at a 2 × 2 kW level. In 2023, we first applied variable core active fibers (VCAF) to a BOFL [54]. The structure of this laser was basically consistent with that in Ref. [47], with the difference being the core device. The active fiber was a spindle-shaped ytterbium-doped fiber with a core/cladding diameter distribution of 25/400 µm–37.5/600 µm–25/400 µm and a total length of 24 m. Obviously, an increase in the core diameter was beneficial for suppressing SRS. According to Ref. [55], a non-wavelength stabilized 976 nm LDs had a higher TMI threshold than a wavelength stabilized 976 nm LDs, so in order to avoid the decrease in the TMI threshold caused by the increase of fiber core, we used a non-wavelength stabilized 976 nm LDs as the pump source of the laser and replaced the combiner with two (18 + 1) × 1 pumps and a signal combiner. Finally, we achieved a 2 × 3 kW laser output, with no SRS observed in the output spectra at both ends. The beam quality at both ends at maximum output power is $M_{A}^{2}=1.98,M_{B}^{2}=2.38$ . The main results are listed in Figure 11. Due to limited pump power, the output power cannot be further improved. In 2023, we achieved the first 2 × 4 kW laser output based on a DCYDF with a core/cladding diameter of 30/400 µm [53]. The corresponding results are shown in Figure 9. In this work, we compared the output characteristics of lasers under different coiling methods and coiling diameters and finally achieved a total power of 8169 W (with output powers of 3769 W and 4400 W at both ends, respectively) under the “8-shaped” coiling method with a maximum coiling diameter of 15.0 cm. Comparative experiments have shown that the BOFL has a better TMI suppression ability compared to the UOFL.

#### 3.1.2. The 2 × 5 kW BOFL-OS Employing 30/600 µm DCYDF

**Figure 12:**
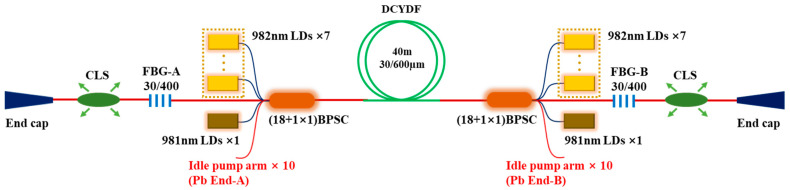
A BOFL-OS based on 30/600 µm DCYDF.

From the current level of device technology, the pumping capacity of fiber combiners based on a cladding of 400 µm is mostly between 5 kW–6 kW. Therefore, without SRS or TMI limitations, it is also impossible to achieve laser output above 2 × 5 kW. Therefore, we considered combining a DCYDF with a core/cladding diameter of 30/600 µm and a (18 + 1) × 1 backward pump and signal combiner with a signal input fiber of 30/600 µm for the BOFL-OS experiment. The structure of the laser is shown in Figure 12. The total length of the active fiber was 40 m, and a wavelength stabilized 982 nm LDs was used as the pump source of the laser. Due to the limited number of pump sources, an additional set of the wavelength stabilized 981 nm LDs was added at both ends to ensure sufficient pump power. The combiner was placed in the resonant cavity, and two OCFBGs were fused with the signal output fibers of the two combiners. The central wavelength of the grating was about 1070 nm, with a bandwidth of about 2.0 nm and reflectivity of 9.1% (FBG-A) and 11% (FBG-B), respectively.

**Figure 13:**
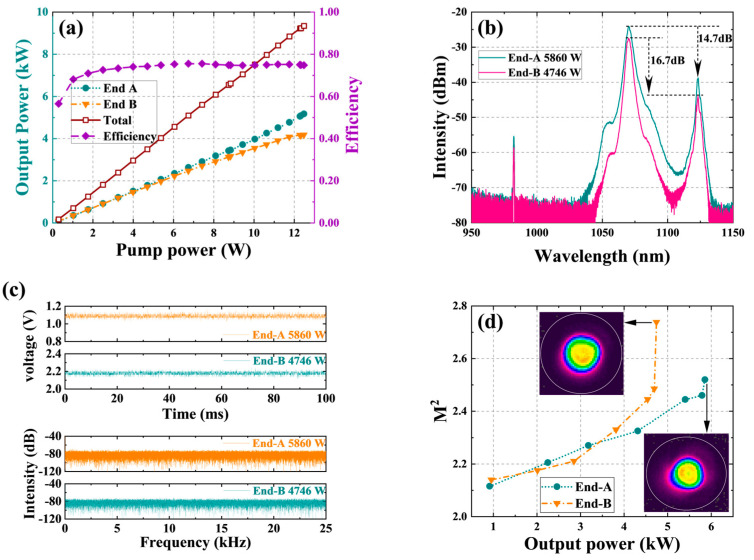
The main results of a BOFL-OS based on 30/600 µm DCYDF. (**a**) The output power and efficiency vary with the pump power; (**b**) the output spectrum at the maximum output power; (**c**) the PD signal and corresponding FFT result of the BOFL at maximum output power; (**d**) evolution of beam quality during the experimental process.

The experimental results are shown in Figure 13. When the pump power at end A and end B was 6508 W and 6592 W, respectively, the output power at both ends reached 5860 W and 4746 W, and the total optical conversion efficiency was 81.0%. The beam quality of the output laser at both ends was $M_{A}^{2}=2.49$ and $M_{B}^{2}=2.48$ . Due to a ~2% difference in reflectivity between the gratings at both ends, the output power at end A was higher than that at end B. At the maximum output power, TMI did not appear in the laser, but the strong SRS limited further improvement in the output power. On the spectrum, the SRS intensity at both ends was 14.7 dB and 16.7 dB lower than the signal, respectively. To our knowledge, this is currently the highest output power of BOFL.

#### 3.1.3. Summary

From the results, through continuous optimization of optical fibers and pump sources, the BOFL-OS have gradually achieved two ports of 2–5 kW laser output. At the same time, it can also be seen that the output beam quality of the lasers with a dual output of more than 3 kW was generally not good enough. As the output power increased, the influence of SRS mutual feedback at both ends of the BOFL-OS also became increasingly significant. This limits the fiber length in the BOFL-OS and has a certain impact on achieving higher output power based on pump optimization. Therefore, further improvement of the output power of the BOFL-OS needs to focus on the suppression of SRS mutual feedback.

### 3.2. High Average Power BOIFL-OA

**Figure 14:**
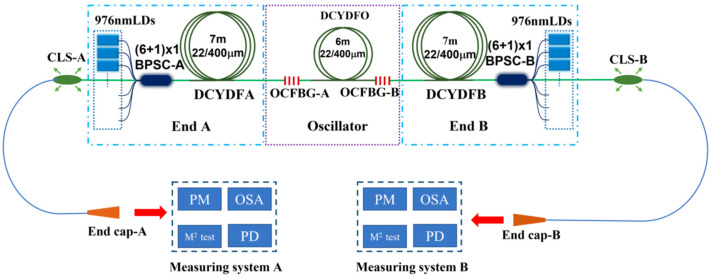
Schematic diagram of a 2 × 2 kW near single-mode BOFL-OA [57].

Due to limitations in the TMI and SRS, it is currently difficult for a BOFL-OS to achieve a high beam quality laser output of over 3 kW at both ends. In 2018, researchers from the Iranian National Center for Laser Science and Technology proposed a new type of oscillating–amplifying integrated fiber laser, which can balance the advantages of fiber laser oscillators and amplifiers, including anti-reflective reflection, high efficiency, and simple control logic [56]. In 2023, we first proposed a BOFL-OA and achieved 2 × 2 kW near single-mode laser output [57]. Figure 14 shows a schematic diagram of the structure of a 2 × 2 kW BOFL-OA. Compared with a typical BOFL-OS, the main difference in its structure is that it divides the active fiber of the laser into three sections, and the grating is located on both sides of the middle section of the fiber. The laser’s output from both ends shares a common resonant cavity, but each has a different amplifying section.

From the overall structure, the BOFL-OA has a longer active fiber, which can provide sufficient pump absorption and improve the efficiency of the laser, and with the advantage of flexible and adjustable output power of two ports, the application prospects are more extensive. In addition, side pumped and signal combiners and chirped and tilted fiber Bragg grating can be introduced between the oscillating section and the amplifying section at both ends of the laser, which plays an important role in suppressing the temporal stability and SRS during laser operation. Here, we will introduce the experimental results of a BOFL-OA with an output power of 2 × 3 kW~4 kW.

#### 3.2.1. A 2 × 3.7 kW BOFL-OA Based on SRS Mutual Feedback Suppression and Pump Wavelength Optimization

**Figure 15:**
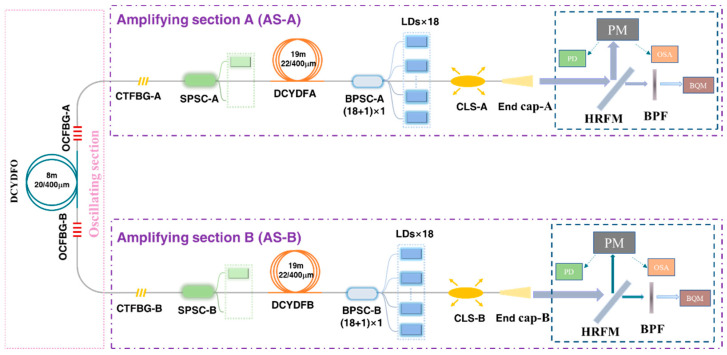
Schematic diagram of a 2 × 3 kW BOFL-OA based on SRS mutual feedback suppression and pump wavelength optimization [58].

**Figure 16:**
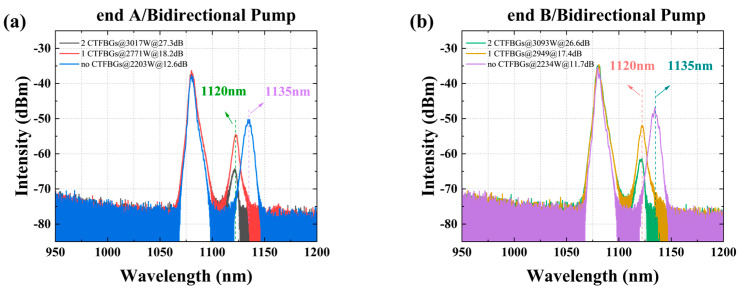
Comparison of output spectra with different numbers of CTFBG under the consistent bidirectional pumping. (**a**) output spectra at end A. (**b**) output spectra at end B [58].

Due to the limitations of TMI, it is difficult to achieve a laser output above 3 kW at both ports based on double-cladding ytterbium-doped fibers with a core-cladding diameter of 22/400 µm and wavelength stabilized 976 nm LDs. In 2023, we achieved a 2 × 3 kW laser output based on the same fiber and a non-wavelength stabilized 976 nm LDs [58]. The maximum output power at the two ports were 3133 W and 3213 W, respectively, and the beam quality was $M_{A}^{2}=1.3$ and $M_{B}^{2}=1.4$ . The structure of the laser is shown in Figure 15. Compared with the basic structure in Figure 14, the main optimization in the current laser lies in two side pumped and signal combiners (SPSC-A and SPSC-B) and two chirped and tilted fiber Bragg gratings (CTFBG-A and CTFBG-B). The function of the SPSC is to directly provide pump power to the oscillating section, enabling the laser to quickly reach a stable state. The function of a CTFBG is to suppress SRS mutual feedback within the laser, as shown in Figure 16. When there was no CTFBG, the SRS intensity was 12.6 dB and 11.7 dB lower than the signal when the output power at both ends reached 2203 W and 2234 W, respectively. After adding a CTFBG at both ends, the SRS intensity was 27.3 dB and 26.6 dB lower than the signal light at output powers of 3017 W and 3093 W, respectively. Due to the presence of a CTFBG, the Stokes light intensity near the center wavelength of 1135 nm decreased, with a peak wavelength of 1120 nm. This result indicates that for a BOFL-OA, due to the long total fiber length, the SRS at both ports is prone to mutual feedback. Introducing SRS filtering devices into the laser is crucial for achieving a higher-power laser output.

**Figure 17:**
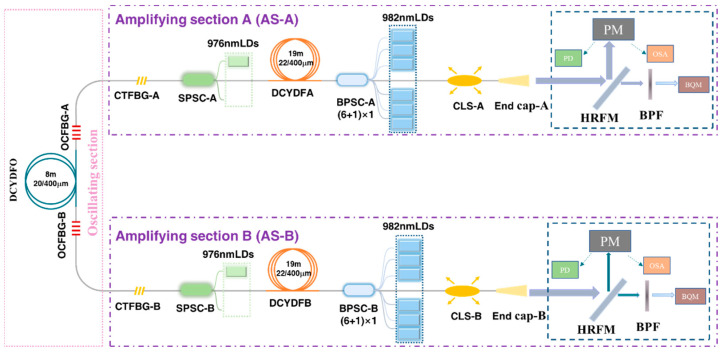
Schematic diagram of a 2 × 3.7 kW BOFL-OA pumped by a wavelength stabilized 982 nm LDs.

**Figure 18:**
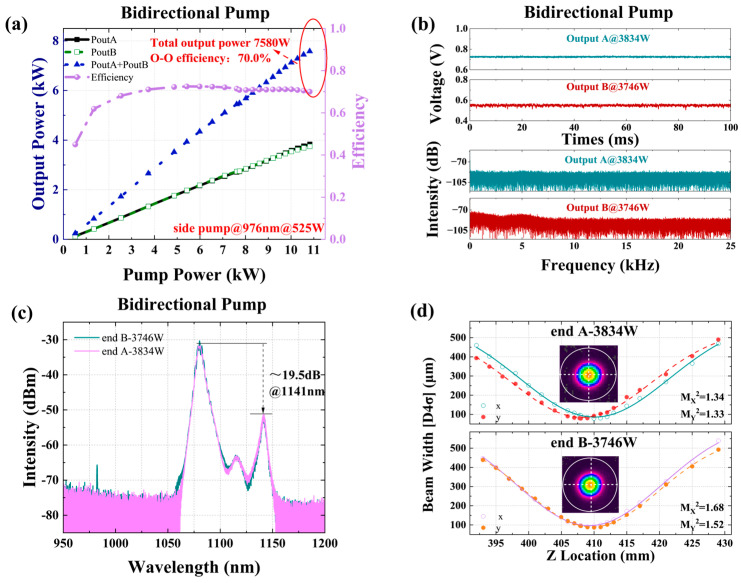
Experimental results of a 2 × 3.7 kW BOFL-OA based on a wavelength stabilized 982 nm LDs. (**a**) The output power and efficiency vary with the pump power; (**b**) the PD signal and corresponding FFT result of the BOFL at maximum output power; (**c**) the output spectrum at the maximum output power; (**d**) the beam quality at the maximum output power.

Due to the limitation of the total pump power and TMI of the non-wavelength stabilized 976 nm LDs, it is difficult to improve the output power of the fiber laser further based on Figure 15. Therefore, based on the above structure, the pump source was replaced with a wavelength stabilized 982 nm LDs, and CTFBG-A was replaced with a new one with a signal wavelength of 1070 nm and a suppression range of 1123 ± 10 nm. The structure and results are shown in Figure 17 and Figure 18, respectively; a maximum output power of 7580 W (A end 3834 W, B end 3746 W) at a total pump power of 10,826 W was achieved, with an efficiency of 70.0%. At this point, obvious TMI features were observed on both ends. Thanks to the presence of two CTFBGs, the Stokes light with center wavelengths of 1123 nm and 1135 nm was suppressed, and the Stokes light with center wavelengths of 1140 nm was amplified. The beam quality at the maximum output power was $M_{A}^{2}=1.33$ and $M_{B}^{2}=1.60$ , respectively. From the results, it can be seen that the current laser was limited by both TMI and SRS, and the output power had reached its limit.

#### 3.2.2. A 2 × 4 kW near Single-Mode BOFL-OA Based on Fiber and Pump Optimization for Balancing TMI and SRS

**Figure 19:**
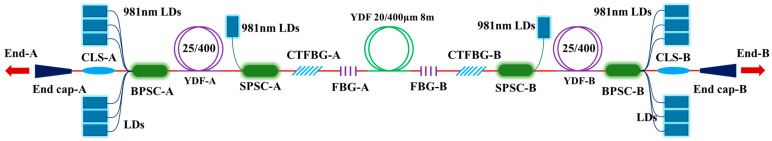
Schematic diagram of a 2 × 4 kW near single mode BOFL-OA based on low absorption fiber and wavelength stabilized 981 nm LDs.

Figure 19 shows a schematic diagram of a BOFL-OA based on a low absorption 25/400 µm DCYDF. The pump absorption coefficient of the active fiber for pump light with a center wavelength of 976 nm was about 1.07 dB/m, which was much lower than the commercial fiber’s ~1.68 dB/m. Reducing the pump absorption coefficient of the fiber was beneficial for improving the TMI threshold of the laser. At the same time, in order to avoid strong SRS caused by excessively long fiber, LDs with a center wavelength of 981 nm was used as the pump source, which has a higher absorption coefficient than a pump source with a center wavelength of 982 nm. The combination of a low absorption fiber and wavelength stabilized 981 nm LDs can balance TMI and SRS. Except for the pump source and active fiber, the rest are consistent with Figure 17.

**Figure 20:**
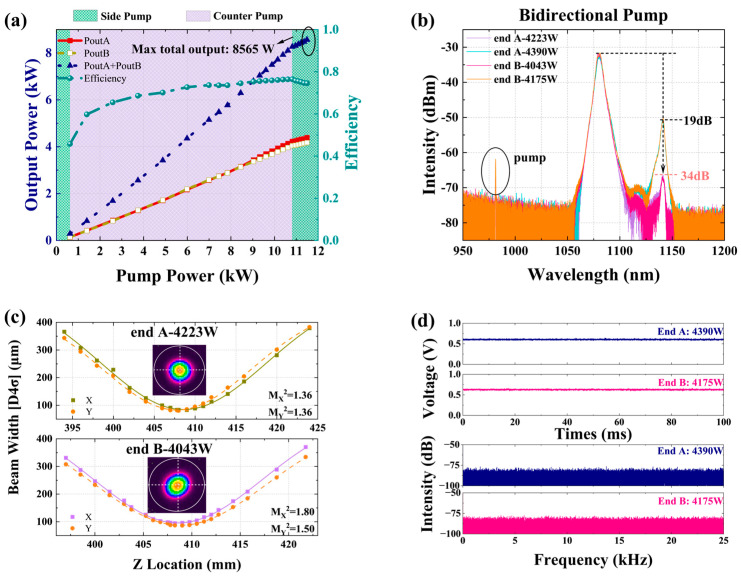
Experimental results of a 2 × 4 kW near single mode BOFL-OA based on low absorption fiber and wavelength stabilized 981 nm LDs. (**a**) The output power and efficiency vary with the pump power; (**b**) the output spectrum at the maximum output power; (**c**) the beam quality at the maximum output power; (**d**) the PD signal and corresponding FFT result of the BOFL at maximum output power.

The results are shown in Figure 20. At a total pump power of 11489 W, we achieved a laser output of 8565 W, with an output power of 4390 W at the end A and 4175 W at the end B, and an optical conversion efficiency of 74.5%. At the highest output power, the SRS intensity of the laser output at both ends of the laser was only 19 dB and 18 dB lower than the signal, respectively. With only a slight increase in output power, the SRS intensity was significantly enhanced, indicating that there is still a phenomenon of SRS mutual feedback, which is due to the insufficient suppression bandwidth of the current CTFBG. From the output signal of the PD and its corresponding FFT results, no TMI was observed at both ports, and the beam quality at both ports was measured as $M_{A}^{2}\sim 1.39$ and $M_{B}^{2}\sim 1.67$ . This is currently the highest output power reported for the BOFL-OA.

#### 3.2.3. A 6 kW Fiber Laser Based on a 2 × 3 kW BOFL-OA through Power Combining

**Figure 21:**
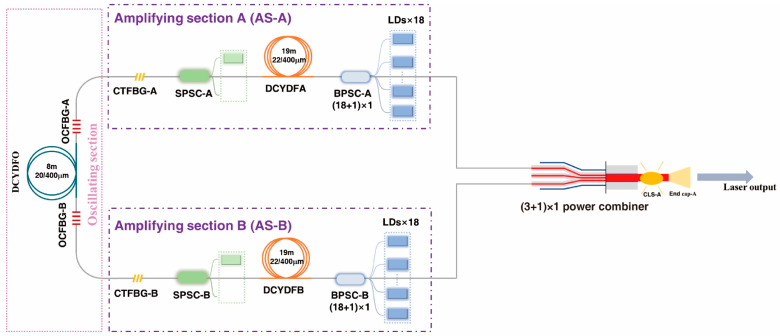
Schematic diagram of a 6 kW fiber laser through power combining based on a 2 × 3 kW BOFL-OA beam combination.

The implementation of a single aperture high-power fiber laser based on BOFL power combining is an important application of the BOFL. On the basis of the 2 × 3 kW BOFL-OA shown in Figure 15 in Section 3.2, a single aperture 6 kW laser was achieved by combining with a self-developed power combiner. The structure of the laser is shown in Figure 21. The main part of the laser is consistent with Figure 15, and the output fibers at end A and end B are fused with the two signal input fibers of the 3 × 1 power combiner (PC). In order to achieve matching with the output fiber, the core/cladding diameter of the signal input fiber of the PC is 25/125 µm, and the core/cladding diameter of the signal output fiber is 50/250 µm. The output laser is fed into the measuring system through size-matched passive fibers and end caps to monitor power, spectrum, and beam quality.

**Figure 22:**
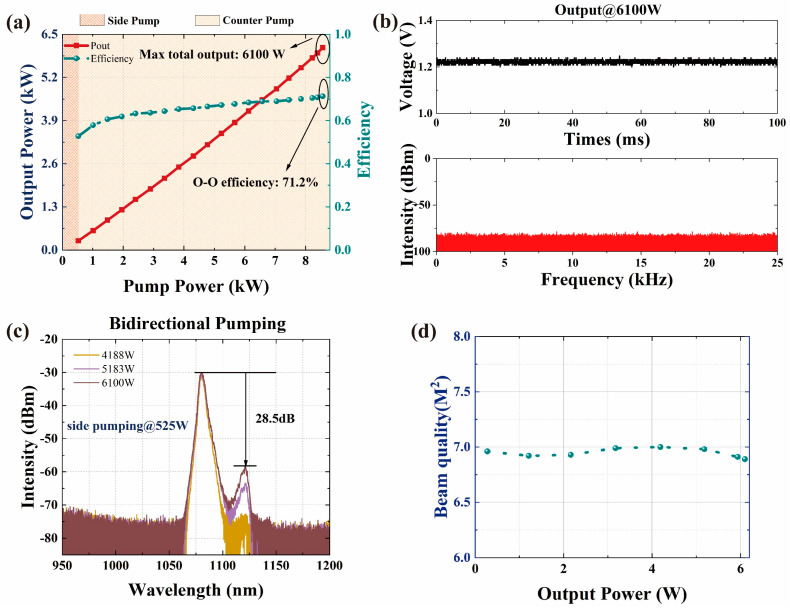
The main results of a 6 kW fiber laser based on a 2 × 3kW BOFL-OA through power combining. (**a**) The output power and efficiency vary with the pump power; (**b**) the PD signal and corresponding FFT result at maximum output power; (**c**) the output spectrum at the maximum output power; (**d**) evolution of beam quality during the experimental process.

The experimental results are shown in Figure 22. According to the results in Section 3.2, the laser can achieve a total power of 6346 W (3133 W of end A and 3213 W of end B) under a bidirectional pump configuration. After the power combining, under the same bidirectional pump configuration as before, the output power of the laser was 6100 W and the combining efficiency was 96%. The SRS intensity at the maximum output power was about 28.5 dB lower than that of the signal, and there was not much difference compared to the results at bidirectional output (end A-27.6 dB, end B-28.1 dB). The beam quality of the output laser deteriorated significantly after passing through the PC. Taking the end A pump as an example, before entering the PC, the beam quality at an output power of 3133 W was $M_{A}^{2}\sim 1.33$ . However, after passing through the PC, the beam quality deteriorates to $M_{A}^{2}\sim 7.10$ . The reason is that the PC’s beam quality maintenance ability is not very good. Figure 22d shows the variation of output laser beam quality with increasing output power, and the beam quality at the maximum output power was $M_{A}^{2}\sim 6.87$ .

#### 3.2.4. Summary

Compared to the BOFL-OS, the BOFL-OA demonstrates stronger power scaling ability, making it easier to achieve high beam quality and higher power laser output. The reason is that the correlation between the two output ports of the laser is reduced, providing more optimization space. Unlike the BOFL-OA, the suppression of TMI in the BOFL-OA largely depends on the TMI threshold of the fiber of the amplifying section. Therefore, more attention should be paid to the optimization of the amplifying section of the fiber and the pump source. In addition, it is known from existing reports that it is crucial to use SRS suppression devices such as CTFBG to suppress the SRS mutual feedback at both ends of the BOFL-OA. On this basis, using fibers with a higher TMI threshold and sufficient backward pump power in the amplifying section of the laser can achieve higher power laser output.

## 4. Experimental Study on High Peak Power QCW BOFL-OS

### 4.1. Experimental Study on High-Power QCW BOFL-OS

Thanks to the unique advantages of QCW fiber lasers in many fields, the development of high beam quality and high peak power QCW fiber lasers has great potential. Compared to high average power CW fiber lasers, QCW fiber lasers have higher peak power, making them more prone to generating SRS and resulting in limited output power. Using fibers with larger cores can, to some extent, suppress SRS, but it can also lead to degradation of beam quality. According to the simulation results in Section 2.2.2 and experimental results in Section 2.3.1, the BOFL has better SRS suppression capability than a UOFL. Therefore, a QCW BOFL can be designed based on fibers with smaller core diameters to achieve higher laser output power while ensuring good beam quality.

**Figure 23:**
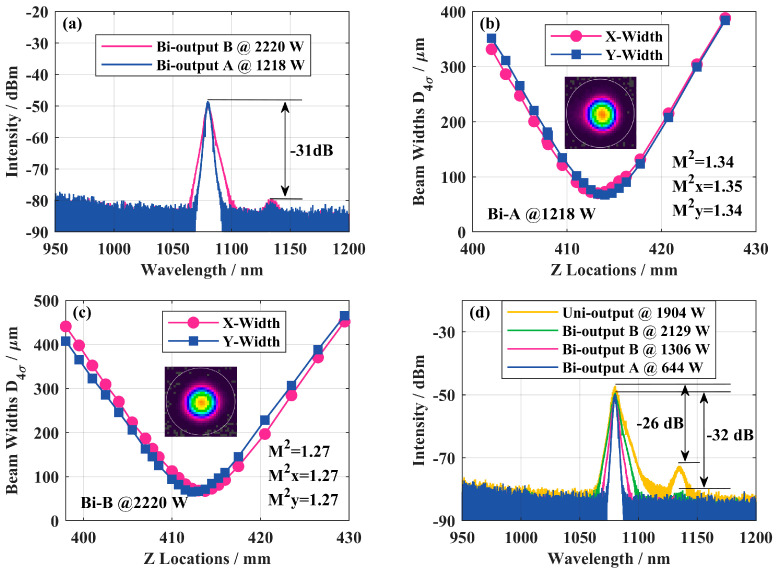
Experimental results of a QCW BOFL. (**a**) The output spectrum at the maximum output power; (**b**) beam quality at end A; (**c**) beam quality at end B; (**d**) comparison of output spectra between a BOFL and UOFL [52].

Figure 6 shows the schematic diagram of the structure of a QCW BOFL-OS [52]. Based on this structure, the QCW BOFL-OS has been validated for the first time. The results are shown in Figure 23. When the total average pump power was 533 W, a laser output with a total average power of 321 W was achieved, corresponding to a total peak power of 3438 W, with a peak power of 1218 W at end A and 2220 W at end B. At the maximum output power, SRS begins to appear on the spectrum, with an intensity of about 31 dB lower than the signal. Due to the small core diameter of the active fiber, the output laser maintains near single-mode beam quality. On this basis, the suppression effect of SRS was also compared with that of a UOFL after replacing the OCFBG at end A with a HRFBG. The results are shown in Figure 7 and Figure 23d, and are described in detail in Section 2.3.1. For a UOFL, the SRS intensity is only 26dB lower than the signal when the output power reaches 1904 W. Whether from the perspective of the output power of the single ports or the total output power of all ports, QCW BOFL-OS have achieved better SRS suppression at higher power levels and have better power enhancement capabilities.

**Figure 24:**
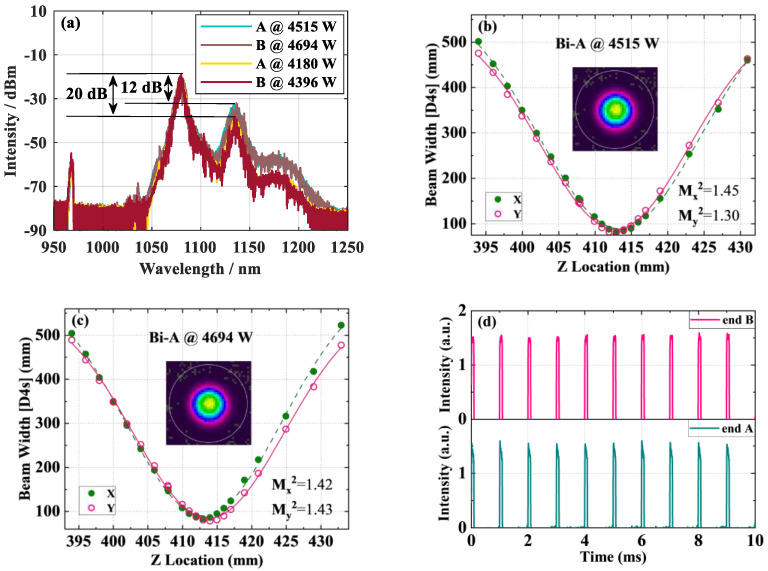
Experimental results of a QCW BOFL based on 25/400µm DCYDF: (**a**) The output spectrum at the maximum output power; (**b**) beam quality at end A; (**c**) beam quality at end B; (**d**) PD output signal.

From the results of Figure 23a, it can be seen that there is already a significant SRS at the current power level, and due to the limitations of the pump power, it is difficult to achieve a significant increase in the output power of the current laser. Considering that the relatively low average output power of a QCW fiber laser, the influence of TMI can be ignored. Therefore, it was considered to use a DCYDF with a core/cladding diameter of 25/400 µm combined with a wavelength stabilized 976 nm LDs to achieve higher output power. The overall structure of the laser and the modulation parameters of the pump source remain unchanged from Figure 6. The total length of active fiber was 20 m. The size of the fiber device matches the active fiber, with an average reflectivity of ~5% and a 3 dB bandwidth of ~1 nm. As shown in Figure 24, at a total average pump power of 1045 W, a laser output with a total average power of 823 W was achieved, with an efficiency of 79%. The total peak power calculated based on the PD signal is 9209 W (4515 W at end A and 4694 W at end B). At the maximum output power, strong SRS appears in the spectrum, with intensities of 12 dB and 20 dB lower than the signal, respectively. Compared to active fibers with a core/cladding diameter of 20/400 µm, the beam quality slightly deteriorated but also maintained near single-mode. Table 6 shows the structural parameters and results comparison of the current QCW BOFL-OS and QCW UOFL. It can be seen that the SRS suppression capability is a significant advantage of QCW BOFL-OS, which can achieve higher beam quality and higher peak power laser output with a smaller core diameter.

### 4.2. New Phenomenon of TMI Suppression in CW + QCW Operation Mode

In fact, the essence of QCW fiber lasers is pump modulation. According to existing reports, pump modulation can improve the TMI threshold of lasers. On the basis of traditional pump modulation and QCW fiber laser, we propose a BOFL-OS with CW + QCW operation mode, which is a pulsed fiber laser with a DC substrate. Under this new structure, it has been verified that adding a QCW pump based on high average power CW operation can improve the TMI threshold of the laser.

**Figure 25:**
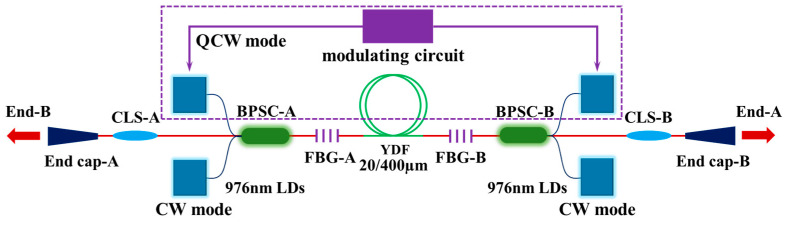
Schematic diagram of the BOFL-OS in CW + QCW operating mode.

**Figure 26:**
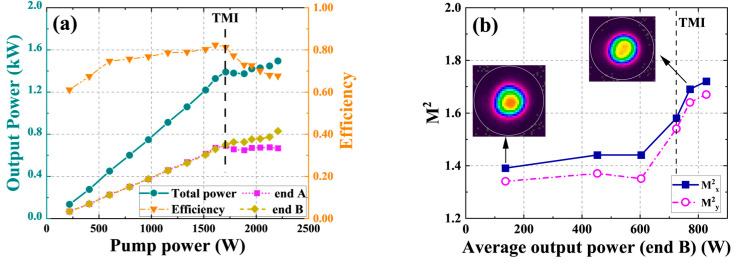

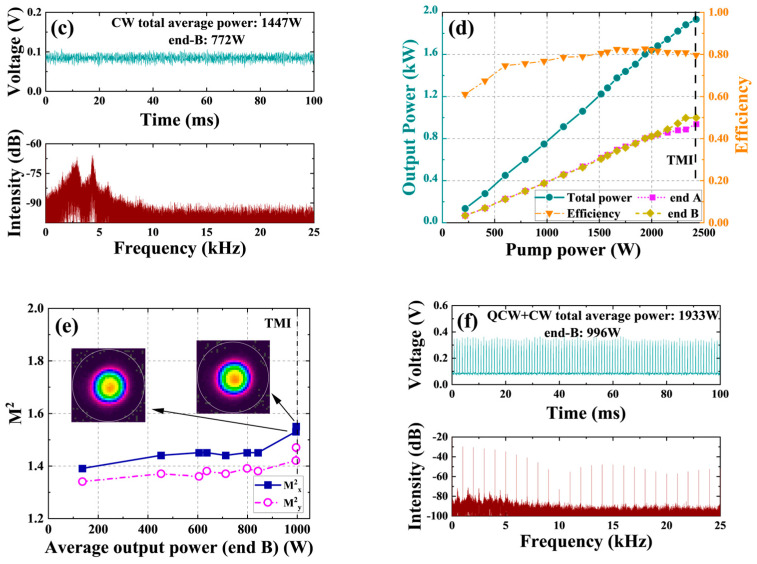
The main experimental results of a QCW + CW BOFL. (**a**) The variation of output power and efficiency with pump power in CW mode; (**b**) the beam quality at the end B under different output powers in the CW mode; (**c**) the PD signal and its corresponding FFT result after TMI appears in the CW mode; (**d**) the variation of output power and efficiency with pump power in the QCW + CW mode. (**e**) The beam quality at the B-end under different output powers in the QCW + CW mode; (**f**) The PD signal and its corresponding FFT result after TMI appears in the QCW + CW mode.

The laser structure is shown in Figure 25. The active fiber and fiber grating used in the laser are consistent with the 2 × 4.5 kW BOFL-OS in Section 4.1. In terms of pump sources, the pump sources were reorganized, with four operating in CW mode and six operating in QCW mode. The modulation frequency of the pump source was 1 kHz, and the pulse duty cycle was 10%. The experimental results are shown in Figure 26. In CW mode, when the pump power was 1711 W and the output power was 1390 W. Prior to this, there was a good linear relationship between output power and efficiency, with a slope efficiency of approximately 85%. Subsequently, as the pump power increased, the increase in output power decreased, and the slope efficiency decreased to 21%. In the PD signal, obvious TMI features were observed, accompanied by degradation of beam quality. The TMI threshold of the current laser was approximately 1390 W. When retesting, we first increased the continuous pump source to an output power of 1223 W, which was close to the TMI threshold of the laser in CW operating mode. Subsequently, we began to increase the QCW pump source. As shown in Figure 26d–f, when all QCW pump sources operated at their maximum output power, the average output power of the laser was 1681 W without TMI. Then, the CW pump source of the laser was further increased. When the total output power reached 1933 W, TMI features were observed in the FFT results of the PD signal, which were manifested as characteristic peaks outside the QCW characteristic frequency in the FFT results. Comparing the experimental results before and after, the addition of the QCW pump significantly improves the average power corresponding to the occurrence of TMI in the laser (an increase of 39%), optimizing both the output laser power and beam quality. When TMI occurred after adding the QCW, the CW pump power was 1887 W, which was also higher than the pump power when TMI occurred only with the CW pump. Therefore, the combination of QCW and CW had a significant effect on suppressing the TMI in lasers. Meanwhile, this is also the first time a BOFL-OS combining CW and QCW has been achieved, with an average power of 1933 W, CW power of 1475 W, and peak power of 7736 W.

### 4.3. Summary

Consistent with the UOFL and CW BOFL, the QCW BOFL can achieve higher peak power and higher beam quality laser output based on fibers with smaller core diameters, thanks to the better SRS suppression ability of BOFL. Moreover, because the actual fiber length is not too long, the output laser’s temporal characteristics at both ports remain consistent, and a single fiber high peak power laser can be achieved through power combining. The novel CW + QCW operating mode has important applications in many fields, and the research found that the suppression effect of the CW + QCW mode on the TMI provides a way to improve the average output power of the laser.

## 5. Conclusions

Table 7 shows the main output results of the BOFL mentioned in the article. Compared to a UOFL, a BOFL can reduce the volume and weight of the system, saving costs. More importantly, it is conducive to suppressing TMI and nonlinear effects represented by SRS and achieving an increase in the output power of single-fiber lasers. At present, the operating mode of BOFL has been extended from CW mode to QCW mode and CW + QCW mode. The BOFL-OS has achieved a total power of >10 kW (5 kW on each port), the BOFL-OA has achieved a total power output of >8 kW (4 kW on each port), and the QCW BOFL-OS has achieved a total peak power output of >9 kW (4.5 kW on each port). From the results, it can be seen that various types of BOFL face different challenges. Overall, TMI and SRS are the main factors limiting the power improvement of high-power fiber lasers. BOFL with different operating modes can achieve a balance between TMI and SRS by combining pump wavelength, pump configuration, fiber coiling, and fiber structure optimization, thereby improving power [51,54].

For BOFL-OS, power improvement is often achieved by sacrificing beam quality. For lasers with a total output of over 6 kW, the beam quality of each port is generally above 2.0, and reducing the fiber diameter will result in SRS enhancement. Therefore, for BOFL-OS, the key lies in optimizing the fiber, pump source, and passive fiber components to improve the beam quality of the output laser. In addition, as the output power increases, it is necessary to introduce SRS suppression devices such as CTFBG to eliminate the mutual feedback of SRS transmitted in different directions.

For BOFL-OA, it is relatively easy to achieve higher power and high beam quality laser output. In addition to balancing TMI and SRS using pump wavelength, pump configuration, fiber coiling, and fiber structure optimization, the phenomenon of SRS mutual feedback is more pronounced than conventional UOFL and BOFL-OS due to the longer total fiber length. Therefore, using SRS suppressor devices such as CTFBG or long-period gratings to isolate SRS mutual feedback is crucial for improving the power of BOFL-OA. It is necessary to focus on increasing the suppression ratio and bandwidth of the devices. In addition, a large part of the output power improvement of the BOFL-OA relies on the backward pump power. Therefore, developing high brightness LD to enhance the pump capacity of the laser is also necessary.

For QCW fiber lasers, SRS remains the main limiting factor due to their high peak power. On the premise of maintaining good beam quality, fiber structure design, pump source optimization, fiber coiling optimization, etc., are still the key to improving output power. From the comparison between the BOFL-OS and the BOFL-OA, it can be seen that if the SRS mutual feedback can be well suppressed, then for the BOFL-OA, the output lasers at both ports are mainly backward pumped, which is conducive to the suppression of SRS. Therefore, the structure can be improved to a QCW BOFL-OA, achieving a further increase in output power.

For the new type of laser with CW + QCW operating mode, a new way to suppress TMI has been discovered, and the next development needs to be carried out from both theoretical and experimental aspects. In theory, it is necessary to study the physical mechanism of TMI suppression in this operating mode and apply it to conventional high-power fiber lasers. In experiments, the effects of the ratio of CW output power and QCW output power, as well as the duty cycle and repetition frequency of QCW output power on output characteristics, are studied. On the basis of the above research, we are moving towards the goal of achieving higher-power fiber lasers.

## Figures and Tables

**Table 1:** The parameters in the theoretical model and their corresponding physical meanings.

Parameter	Symbol	Parameter	Symbol
$\alpha_{m}^{p}$	Pump light loss coefficient	$\Gamma_{p}$	Pump light filling factor
$\alpha_{n}^{s}$	Signal light loss coefficient	$\Gamma_{s}$	Signal light filling factor
$\sigma_{m}^{ep}$	Pump light emission cross-section	$N_{0}$	Total doping ion concentration
$\sigma_{m}^{ap}$	Pump light absorption cross-section	$N_{1}$	Ground state doping ion concentration
$\sigma_{n}^{es}$	Signal light emission cross-section	$N_{2}$	Excited state doping ion concentration
$\sigma_{n}^{as}$	Signal light absorption cross-section	$A_{eff}$	Effective area of the fiber core
$\tau_{1}=1/\Omega_{R}$	Reciprocal of quartz molecular oscillation frequency	τ	Upper-level lifetime
$\tau_{2}$	Damping vibration time	$\gamma_{k}$	Nonlinear parameters
$f_{R}$	Delayed Raman response		

**Table 2:** The main parameters and their values in simulation.

Parameters	Value
Signal wavelength	1080 nm
Pump wavelength	976 nm
Total pump power	4000 W (2000 W × 2 for bidirectional pump)
Pump absorption coefficient	1.07 dB/m @ 976 nm
Core diameter of YDF	20 µm
Inner-cladding diameter of YDF	400 µm
Length of YDF	20 m
3 dB bandwidth of FBG-A	1 nm
3 dB bandwidth of FBG-B	1 nm
Reflectivity of FBG-A	10% or 99%
Reflectivity of FBG-B	10%

**Table 3:** The total power and Raman power in the output laser of the BOFL and UOFL under different pump configurations.

Laser Type	Pump Configuration	Total Power/W	Raman Power/W
BOFL	End A pump 4000 W	3523	1.073
	Bidirectional pump 2000 W × 2	3526	0.390
	Bidirectional pump 4000 W × 2	7056	39.421
UOFL	Forward pump 4000 W	3374	206.52
	Backward pump 4000 W	3442	10.600
	Bidirectional pump 2000 W × 2	3408	31.666

**Table 4:** The maximum temperature of the fiber core and coating in the BOFL and UOFL under different pump configurations.

Laser Type	Pump Configuration	Maximum Temperature of Fiber Core	Maximum Temperature of Coating
BOFL	End A pump 4000 W	112.10 °C	48.31 °C
	Bidirectional pump 2000 W × 2	78.91 °C	37.82 °C
	Bidirectional pump 4000 W × 2	120.72 °C	50.62 °C
UOFL	Forward pump 4000 W	112.65 °C	48.46 °C
	Backward pump 4000 W	119.50 °C	50.29 °C
	Bidirectional pump 2000 W × 2	78.63 °C	39.35 °C

**Table 5:** The TMI thresholds for the BOFL and UOFL based on 25/400 µm YDF under the same conditions.

Structure	Pump Distribution	TMI Threshold (W)	Pump Power (W)
UOFL	Bidirectional pump (1:1)	1910	2820
BOFL	Bidirectional pump (1:1)	2515	3646

**Table 6:** Comparison of main results of the current QCW BOFL-OS and QCW UOFL.

Laser Type	Fiber Type	Fiber Length	Pump Wavelength	Peak Power		M^2^	SRS
QCW UOFL [48]	30/400 µm YDF	15 m	976 nm	9713 W		2.29	>25 dB
QCW UOFL [49]	Spindle-shaped YDF	24 m	976 nm	6491 W		1.38	20.3 dB
QCW UOFL [50]	Spindle-shaped YDF	24 m	976 nm	7398 W		1.43	26.0 dB
QCW BOFL-OS [52]	20/400 µm YDF	25 m	981 nm	3438 W	1218 W	1.34	31.0 dB
					2220 W	1.27	31.0 dB
QCW BOFL-OS	25/400 µm YDF	20 m	976 nm	9209 W	4515 W	1.37	12.0 dB
					4694 W	1.42	20.0 dB

**Table 7:** The main output results of the BOFL mentioned in the article.

Laser Type	Fiber/µm	Pump Wavelength/nm	Output Power/W		TMI/W	SRS/dB	M^2^
BOFL-OS	20/400	976	3973	1948	>1948	35	1.41
				2025	>2025	35	1.50
	25/400–37.5/600–25/400	Non-wavelength stabilized 976	6105	3265	>3265	>40	1.98
				2840	>2840	>40	2.38
	30/400	981	8169	3769	>3769	>45	2.13
				4400	>4400	>45	2.36
	30/600	982	10,606	5860	>5860	14.7	2.49
				4746	>4746	16.7	2.48
BOFL-OA	22/400 + 22/400	976	3845	1955	>1955	29.1	1.30
				1890	>1890	31.4	1.44
	22/400 + 22/400	Non-wavelength stabilized 976	6346	3133	>3133	21.0	1.33
				3213	>3213	20.6	1.41
	22/400 + 22/400	982	7580	3834	>3834	19.5	1.33
				3746	3746	19.5	1.60
	22/400 + 25/400	981	8565	4390	>4390	19.0	1.39
				4175	>4175	19.0	1.67
QCW BOFL-OS	20/400	981	3438	1218	>1218	31.0	1.34
				2220	>2220	31.0	1.27
	25/400	976	9209	4515	>4515	12.0	1.37
				4694	>4694	20.0	1.42

## Data Availability

Data is contained within the article.
